# Histology-Based Induction Chemoradiotherapy Followed by Surgery for Stage IIIA-N2 Non-Small Cell Lung Cancer: A Prospective Observational Study

**DOI:** 10.5761/atcs.oa.26-00024

**Published:** 2026-05-13

**Authors:** Hironori Ishibashi, Ayaka Asakawa, Yusuke Sugita, Yuya Ishikawa, Ryo Wakejima, Takayuki Honda, Yasunari Miyazaki, Ryoichi Yoshimura, Kenichi Okubo

**Affiliations:** 1 Department of Thoracic Surgery, Graduate School of Medical and Dental Sciences, Institute of Science Tokyo, Tokyo, Japan; 2 Department of Respiratory Medicine, Graduate School of Medical and Dental Sciences, Institute of Science Tokyo, Tokyo, Japan; 3 Department of Radiation Therapeutics and Oncology, Graduate School of Medical and Dental Sciences, Institute of Science Tokyo, Tokyo, Japan

**Keywords:** non-small cell lung cancer, prognosis, histology, induction chemoradiation

## Abstract

**Purpose:**

Although immune checkpoint inhibitor-based perioperative therapy has become the standard for resectable stage IIIA-N2 non-small cell lung cancer (NSCLC), not all patients are eligible for this approach. This study evaluated long-term outcomes of histology-based induction chemoradiotherapy (CRT) followed by surgery.

**Methods:**

This prospective observational study enrolled 48 consecutive patients with pathologically confirmed stage IIIA-N2 NSCLC between April 2010 and May 2025. Patients with squamous cell carcinoma (Sq) received cisplatin/vinorelbine, while those with non-squamous cell carcinoma (non-Sq) received cisplatin/pemetrexed, both with concurrent radiotherapy (50 Gy), followed by surgery.

**Results:**

All 48 patients (25 Sq, 23 non-Sq) completed induction CRT; 45 (93.8%) underwent surgery, with 97.8% achieving complete resection. Median follow-up was 48.8 months. The 5-year overall survival/disease-free survival (DFS) rates were 77.9%/77.3% for Sq and 70.5%/33.7% for non-Sq cohorts. Grade 3/4 toxicities were more frequent in Sq patients (80.0% vs. 21.7%, p <0.001). Exploratory analysis revealed inferior DFS in epidermal growth factor receptor (EGFR)-mutated non-Sq patients, though overall survival remained favorable with subsequent tyrosine kinase inhibitor therapy.

**Conclusion:**

Histology-based induction CRT followed by surgery demonstrated feasibility and favorable outcomes, particularly for Sq and EGFR-negative non-Sq NSCLC. These findings provide a reference for patients ineligible for immune checkpoint inhibitor-based therapy.

## Abbreviations

CRT
: chemoradiotherapy
DFS
: disease-free survival
EGFR
: epidermal growth factor receptor
NSCLC
: non-small cell lung cancer
OS
: overall survival
Sq
: squamous cell carcinoma
TKI
: tyrosine kinase inhibitor

## Introduction

Lung cancer remains the leading cause of cancer-related mortality worldwide.^1)^ Non-small cell lung cancer (NSCLC) constitutes approximately 85% of all lung cancer cases, with squamous cell carcinoma (Sq) and adenocarcinoma being the most common histological subtypes.^2)^ Despite advances in early detection and treatment, the prognosis of patients with locally advanced NSCLC, particularly those with mediastinal lymph node involvement (stage III), remains poor, with 5-year survival rates ranging from 15% to 30%.^3)^ The management of stage III NSCLC is particularly challenging owing to its heterogeneity. Multimodal treatment strategies including surgery, chemotherapy, and radiotherapy have been explored to improve outcomes.^4,5)^ Although some studies have reported improved survival with trimodality therapy compared with chemoradiotherapy alone, others have raised concerns about increased toxicity and perioperative complications.^4,6)^ Furthermore, the choice of chemotherapeutic agents during induction has been shown to influence both response rates and toxicity profiles, necessitating careful consideration of histological subtypes when designing treatment regimens.^7,8)^ In recent years, immune checkpoint inhibitor (ICI)-based perioperative therapy has demonstrated remarkable efficacy in resectable NSCLC. The CheckMate 816 (ClinicalTrials.gov identifier: NCT02998528) trial showed superior pathological complete response (pCR) rates and event-free survival with neoadjuvant nivolumab plus chemotherapy compared with chemotherapy alone.^9)^ Subsequently, the KEYNOTE-671 trial (ClinicalTrials.gov identifier: NCT03425643) demonstrated that perioperative pembrolizumab plus chemotherapy significantly improved OS and event-free survival in resectable stage II–IIIA NSCLC.^10)^ Based on these landmark trials, ICI-based perioperative therapy has become the contemporary standard of care for resectable stage III NSCLC in many countries. However, not all patients are eligible for ICI-based therapy due to contraindications such as autoimmune diseases, severe immunosuppression, or organ transplantation. Furthermore, access to ICIs may be limited in certain healthcare settings due to cost or availability constraints. Therefore, understanding the long-term outcomes of alternative treatment strategies remains clinically relevant. Additionally, the optimal regimen for induction therapy in the pre-ICI era, particularly with respect to tailoring treatments based on histological subtypes, has not been fully elucidated. In this study, conducted before the widespread adoption of ICIs, we aimed to evaluate the long-term efficacy and safety of histologically subtype–specific concurrent induction chemoradiotherapy (CCRT), followed by surgery in patients with stage IIIA-N2 NSCLC. We hypothesized that customizing chemotherapy regimens based on histological subtype could optimize treatment outcomes while maintaining a tolerable toxicity profile.

## Materials and Methods

### Study design

This was a prospective, non-randomized observational study conducted from April 2010 to May 2025 at a single tertiary referral center. Consecutive patients with pathologically confirmed stage IIIA-N2 NSCLC were enrolled and treated according to our institutional protocol, which stratified treatment based on histological subtype: squamous cell carcinoma (Sq cohort) and non-squamous cell carcinoma (non-Sq cohort). Patients were assigned to each cohort based solely on histological diagnosis, and no randomization was performed.

### Eligibility criteria

We reviewed the medical records of patients who underwent surgery for primary NSCLC between April 2010 and May 2025 at the Institute of Science in Tokyo, Japan. Perioperative data were collected from our institutional database and electronic medical records, including patient characteristics, tumor characteristics, surgical factors, and postoperative course.

The eligibility criteria included: cytologically or histologically proven NSCLC^11)^; c-stage and p-stage determined according to the 8th edition of the Union Internationale Contre le Cancer-TNM staging system^12)^; resectable tumor at the time of diagnosis, as evaluated by a multidisciplinary group of experienced surgeons, radiation oncologists, medical oncologists, and pneumologists; patient age between 20 and 75 years; Eastern Cooperative Oncology Group performance status of 0–1; patients in a satisfactory medical condition to undergo chemotherapy, thoracic radiotherapy, or surgery; and provision of written informed consent for participation. All clinical and pathological stages were retrospectively reassessed according to the 8th edition of the TNM classification to ensure consistency across the study period. The exclusion criteria encompassed any previous treatment with chemotherapy or thoracic radiotherapy, as well as a history of respiratory failure, cardiac failure, or invasive cancer. Pathological confirmation of N2 disease was obtained in all patients through endobronchial ultrasound-guided transbronchial needle aspiration (EBUS-TBNA), video-assisted thoracoscopic surgery (VATS), or mediastinoscopy.

### Study registration and ethical considerations

This study was initiated in 2010, before the full implementation of mandatory clinical trial registration in Japan. At that time, prospective registration was not uniformly required for all observational studies at our hospital. This study was conducted in accordance with the ethical standards of the Declaration of Helsinki and was approved by the Institutional Review Board (IRB) of the Tokyo Hospital Institute of Science (approval numbers: R2013-010 and R2014-019). Written informed consent was obtained from all participants prior to their participation in this study.

### Induction treatment

**Fig. 1:**
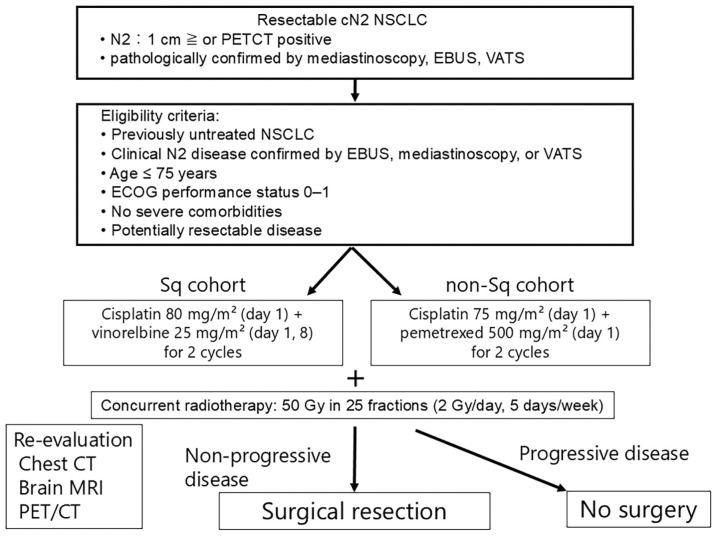
Study flow diagram of the 2 parallel cohorts stratified by histological subtype. NSCLC, non-small cell lung cancer; PET, positron emission tomography; CT, computed tomography; EBUS, endobronchial ultrasound; VATS, video-assisted thoracoscopic surgery; ECOG, Eastern Cooperative Oncology Group; Sq, squamous; MRI, magnetic resonance imaging

After initial workup and multidisciplinary evaluation, patients were assigned to 1 of 2 treatment cohorts according to histological subtype (squamous vs. non-squamous). The Sq cohort received a combination of cisplatin (80 mg/m^2^ on days 1 and 29) and vinorelbine (25 mg/m^2^ on days 1, 8, 29, and 36). The non-Sq cohort received chemotherapy with cisplatin (75 mg/m^2^ on days 1 and 29) and pemetrexed (500 mg/m^2^ on days 1 and 29) (**Fig. 1**). Both cohorts received concurrent radiotherapy. Radiotherapy was delivered using 3-dimensional conformal radiation therapy (3D-CRT) or intensity-modulated radiation therapy (IMRT) techniques, depending on institutional availability during the study period. The clinical target volume included the primary tumor and involved mediastinal lymph nodes with appropriate margins. The planned total radiation dose was 50 Gy delivered in 25 fractions over 5 weeks. This dose was selected based on previous institutional experience and published data suggesting acceptable toxicity profiles with effective tumor downstaging.^13,14)^ For patients who developed progressive disease during induction therapy and were deemed unresectable at reassessment, radiotherapy was escalated to a total dose of 66 Gy with concurrent cisplatin-based chemotherapy as definitive treatment.

### Response evaluation and assessment for surgery

Patients underwent a preoperative staging workup within 2 weeks of completing induction CRT and were reassessed according to the Response Evaluation Criteria in Solid Tumors guidelines.^15)^ Surgical resection with mediastinal lymph node dissection was performed 3 or 4 weeks after induction therapy was completed. For tumors that progressed during the induction treatment and were deemed unresectable at the time of reassessment, patients were administered radiotherapy at a total dose of 66 Gy with concurrent cisplatin-based chemotherapy.

### Surgery

Lobectomy was performed to achieve complete resection of the primary tumor and its extensions, with systematic mediastinal lymph node dissection. The surgical approach (thoracotomy vs. VATS) was selected based on tumor characteristics and surgeon preference. When necessary, bronchoplasty or pulmonary artery reconstruction was performed to avoid pneumonectomy and preserve lung function. pCR was defined as the absence of viable tumor cells in both the resected lung specimen and all examined lymph nodes. Major pathological response (MPR) was defined as ≤10% viable tumor cells in the resected specimen.

### EGFR mutation analysis

Epidermal growth factor receptor (EGFR) mutation status was analyzed retrospectively in available tumor specimens from the non-Sq cohort using standard molecular testing methods. Early cases were tested using polymerase chain reaction–based assays, whereas more recent cases were evaluated using Oncomine. This analysis was performed as an exploratory investigation and was not prespecified in the original study protocol. The results should therefore be interpreted with appropriate caution.

### Endpoints

The primary endpoint was 2-year disease-free survival (DFS), and the secondary endpoints were induction therapy response rate, complete resection rate, overall survival (OS), and incidence of adverse events. Recurrence patterns were classified as locoregional or distant. Locoregional recurrence was defined as recurrence occurring in the ipsilateral lung, bronchial stump, or regional lymph nodes (hilar or mediastinal). Distant recurrence was defined as recurrence occurring outside the thoracic cavity or involving the contralateral organs. When both locoregional and distant recurrence were detected simultaneously, the event was classified as distant recurrence.

Toxicities were determined using the National Cancer Institute Common Toxicity Criteria version 4.0.^16)^ Primary and secondary endpoints were evaluated by an external multidisciplinary monitoring committee.

### Statistical analyses

This observational study was designed to enroll consecutive eligible patients over a 5-year period, with an anticipated enrollment of approximately 35 patients per cohort based on institutional patient volume. Sample size estimation was based on detecting a clinically meaningful improvement in 2-year DFS from a historical benchmark of 40%–60%, with assumed 1-sided alpha of 0.05 and power of 80%. However, due to slower than anticipated patient accrual, the study was closed after enrolling 48 patients in total.

Categorical variables were compared using the chi-squared test or Fisher’s exact test as appropriate. Continuous variables were compared using the Student’s t-test or Mann–Whitney U test. Survival outcomes were estimated using the Kaplan–Meier method, and differences between groups were assessed using the log-rank test. Follow-up time was calculated using the reverse Kaplan–Meier method. All reported p-values are 2-sided, and statistical significance was defined as p <0.05. Statistical analyses were performed using Stata software version 18.0 (Stata Corp LP, College Station, TX, USA).

## Results

### Patient characteristics

Between April 2010 and May 2025, 48 patients were prospectively enrolled, including 25 patients in the Sq cohort and 23 in the non-Sq cohort, including 3 cases of poorly differentiated NSCLC. Baseline patient characteristics are summarized in **Table 1**. The median age of the entire cohort was 69 years. Patients in the Sq cohort were significantly older than those in the non-Sq cohort (p = 0.013). Pathological confirmation of N2 disease was obtained in all 48 patients, and the diagnostic approach for N2 disease did not differ significantly between the 2 cohorts.

**Table 1:** Baseline patient and tumor characteristics

	Sq (n = 25)	Non-Sq (n = 23)	p Value
Sex, n (%)			
Male	18 (72.0%)	14 (60.9%)	0.668
Female	7 (28.0%)	9 (39.1%)	
Age (years), n (%)	69.8 (5.5)	62.8 (12.4)	*0.013*
ECOG performance status, n (%)			
0	22 (88.0%)	22 (95.7%)	0.338
1+	3 (12.0%)	1 (4.3%)	
N2 diagnosis			
EBUS	19 (76.0%)	15 (65.2%)	0.602
VATS	2 (8.0%)	4 (17.4%)	
Mediastinoscopy	4 (16.0%)	4 (17.4%)	
cT			
1a	3 (12.0%)	0 (0.0%)	0.231
1b	3 (12.0%)	5 (21.7%)	
1c	4 (16.0%)	5 (21.7%)	
2a	1 (4.0%)	4 (17.4%)	
2b	3 (12.0%)	4 (17.4%)	
3	7 (28.0%)	4 (17.4%)	
4	4 (16.0%)	1 (4.3%)	
ycN			
N0	14 (56.0%)	11 (47.8%)	0.176
N1	1 (4.0%)	5 (21.7%)	
N2	10 (40.0%)	7 (30.5%)	
ycStage			
IA	10 (40.0%)	5 (21.7%)	0.109
IB	1 (4.0%)	4 (17.4%)	
IIA	1 (4.0%)	0 (0.0%)	
IIB	1 (4.0%)	4 (17.4%)	
IIIA	12 (48.0%)	8 (34.8%)	
IV	0 (0.0%)	2 (8.7%)	
Response rate (%)	49.2 (3.0)	23.5 (3.3)	*<0.001*
Response to induction chemoradiotherapy			
Partial response	23 (92.0%)	7 (30.4%)	*<0.001*
Stable disease	2 (8.0%)	14 (60.9%)	
Progressive disease	0 (0.0%)	2 (8.7%)	

p values under 0.05 are provided in italic.

Sq, squamous cell carcinoma; non-Sq, non-squamous cell carcinoma; ECOG, Eastern Cooperative Oncology Group; VATS, video-assisted thoracoscopic surgery

### Completion of induction chemoradiotherapy and treatment-related toxicity

All 48 patients completed the planned induction chemoradiotherapy protocol. Temporary interruption of radiotherapy due to hematologic toxicity occurred in 25 patients, but all patients ultimately received the planned total radiation dose of 50 Gy. No treatment-related deaths were observed. The incidence of grade 3 or 4 adverse events was significantly higher in the Sq cohort than in the non-Sq cohort (80.0% vs. 21.7%, p <0.001). The most frequent severe adverse event was neutropenia, which occurred in 25 patients (52.1%), including 5 cases complicated by febrile neutropenia (**Table 2**).

**Table 2:** Grade 3–4 toxicities during induction chemoradiotherapy

	Sq (n = 25)	Non-Sq (n = 23)	p Value
Nausea/vomiting			
Grade 3	9 (36.0%)	3 (13.0%)	*0.036*
Grade 4	1 (4.0%)	0 (0%)	
Esophagitis			
Grade 3	3 (12.0%)	1 (4.3%)	*0.187*
Grade 4	1 (4.0%)	0 (0%)	
Pneumonitis			
Grade 3	2 (8.0%)	1 (4.3%)	N/A
Grade 4	0 (0%)	0 (0%)	
Renal dysfunction			
Grade 3	0 (0%)	1 (4.3%)	N/A
Grade 4	0 (0%)	0 (0%)	
Liver dysfunction			
Grade 3	1 (4.0%)	1 (4.3%)	N/A
Grade 4	0 (0%)	0 (0%)	
Anemia			
Grade 3	2 (8.0%)	0 (0%)	N/A
Grade 4	0 (0%)	0 (0%)	
Neutropenia			
Grade 3	4 (16.0%)	2 (8.7%)	*0.002*
Grade 4	15 (60.0%)	4 (17.4%)	
Thrombopenia			
Grade 3	1 (4.0%)	0 (0%)	N/A
Grade 4	0 (0%)	0 (0%)	

Values are presented as the number of patients (%).

p values under 0.05 are provided in italic.

Sq, squamous cell carcinoma; non-Sq, non-squamous cell carcinoma; N/A, not available

### Radiological response to induction therapy

The objective response rate after induction therapy was significantly higher in the Sq cohort than in the non-Sq cohort. In the Sq cohort, 92.0% achieved a partial response with marked tumor shrinkage. In contrast, in the non-Sq cohort, 30.4% achieved a partial response; progressive disease during induction therapy was observed in 2 patients, both of whom developed bone metastases.

### Surgical treatment and perioperative outcomes

After completion of induction therapy and multidisciplinary reassessment, 46 of the 48 patients were considered eligible for surgical resection. One patient declined surgery; hence, 45 patients (93.8%) ultimately underwent radical surgical resection (**Table 3**). The surgical approach was thoracotomy in 38 patients (84.4%), and lobectomy was performed in 36 patients (80.0%), bilobectomy in 5 patients (11.1%), and combined lobectomy plus segmentectomy in 4 patients (8.9%). Bronchial or pulmonary artery reconstruction was performed in 10 patients (22.2%) to avoid pneumonectomy. Complete resection (R0) was achieved in 44 patients (97.8%). Microscopically positive margins (R1) were observed in only 1 patient (2.2%) in the non-Sq cohort. No surgery-related mortality was observed. pCR was achieved significantly more frequently in the Sq cohort (p = 0.002). MPR was also significantly higher in the Sq cohort (p = 0.003).

**Table 3:** Surgical procedures and postoperative outcomes

	Sq (n = 24)	Non-Sq (n = 21)	p value
Sex, n (%)			
Male	18 (75.0%)	14 (66.7%)	0.538
Female	6 (25.0%)	7 (33.3%)	
Age (years), n (%)	69.6 (5.4)	62.1 (12.8)	*0.012*
ECOG performance status, n (%)			
0	21 (95.5%)	20 (87.0%)	0.317
1+	1 (4.5%)	3 (13.0%)	
cT			
1a	3 (12.5%)	0 (0.0%)	0.235
1b	3 (12.5%)	5 (23.8%)	
1c	4 (16.7%)	5 (23.8%)	
2a	1 (4.2%)	4 (19.0%)	
2b	3 (12.5%)	3 (14.3%)	
3	6 (25.0%)	3 (14.3%)	
4	4 (16.7%)	1 (4.8%)	
Approach			
Open thoracotomy	20 (83.3%)	18 (85.7%)	0.826
VATS	4 (16.7%)	3 (14.3%)	
ycN			
N0	14 (58.3%)	9 (42.9%)	0.019
N1	0 (0.0%)	6 (28.6%)	
N2	10 (41.7%)	6 (28.6%)	
Operation time (min), mean (SD)	231.2 (96.0)	241.2 (82.2)	0.709
Bleeding (mL), mean (SD)	273.3 (195.0)	285.1 (200)	0.843
R0	24 (100%)	20 (95.2%)	0.280
Procedure			
Lobectomy	21 (87.5%)	15 (71.4%)	0.376
Bilobectomy	1 (4.2%)	4 (19.0%)	
Lobectomy + segmentectomy	2 (8.3%)	2 (9.6%)	
Response rate (%)	49.2 (3.0)	23.5 (3.3)	*<0.001*
Response to induction chemoradiotherapy			
Partial response	23 (92.0%)	7 (30.4%)	*<0.001*
Stable disease	2 (8.0%)	14 (60.9%)	
Progressive disease	0 (0.0%)	2 (8.7%)	
Plasty			
Total	8 (33.3%)	2 (9.5%)	0.055
PA	3 (12.5%)	0 (0.0%)	
Br	3 (12.5%)	0 (0.0%)	
PA+Br	2 (8.3%)	2 (9.5%)	
Drainage (days), mean (SD)	4.5 (2.4)	4.6 (2.1)	0.881
Postoperative stay (days), mean (SD)	13.3 (1.1)	13.2 (1.7)	0.941
Complication			
Total	9 (37.5%)	7 (33.3%)	0.772
Atelectasis	4 (16.7%)	2 (9.5%)	0.482
Pneumonia	4 (16.7%)	1 (4.8%)	0.205
Air leakage	3 (12.5%)	0 (0.0%)	0.094
Atrial fibrillation	3 (12.5%)	1 (4.8%)	0.363
ypN			
0	15 (62.5%)	7 (33.3%)	0.076
1	0 (0.0%)	2 (9.5%)	
2	9 (37.5%)	12 (57.1%)	
ypStage			
0	11 (45.8%)	1 (4.8%)	*0.023*
IA	4 (16.7%)	6 (28.5%)	
IB	0 (0.0%)	0 (0.0%)	
IIA	0 (0.0%)	0 (0.0%)	
IIB	0 (0.0%)	2 (9.5%)	
IIIA	9 (37.5%)	11 (52.4%)	
IV	0 (0.0%)	1 (4.8%)	
MPR	22 (91.7%)	11 (52.4%)	*0.003*
pCR	11 (45.8%)	1 (4.8%)	*0.002*
Ef			
0	0 (0.0%)	1 (4.8%)	*0.007*
1a	2 (8.4%)	7 (33.3%)	
1b	0 (0.0%)	2 (9.5%)	
2	11 (45.8%)	10 (47.6%)	
3	11 (45.8%)	1 (4.8%)	

p values under 0.05 are provided in italic.

Sq, squamous cell carcinoma; non-Sq, non-squamous cell carcinoma; ECOG, Eastern Cooperative Oncology Group; EBUS, endobronchial ultrasound; VATS, video-assisted thoracoscopic surgery; PA, pulmonary artery; Br, bronchus; MPR, major pathological response; pCR, pathological complete response; yp, post-induction pathological staging; Ef, effect

### Primary endpoint: 2-year DFS

The primary endpoint of 2-year DFS was achieved in 34 of 45 patients who underwent surgery (75.6%). When analyzed by cohort, the 2-year DFS rate was significantly higher in the Sq cohort than in the non-Sq cohort (84.0% vs. 56.5%, p = 0.032), meeting the prespecified threshold of clinical benefit (60%) in the Sq cohort but not in the non-Sq cohort.

### Survival outcomes

**Fig. 2:**
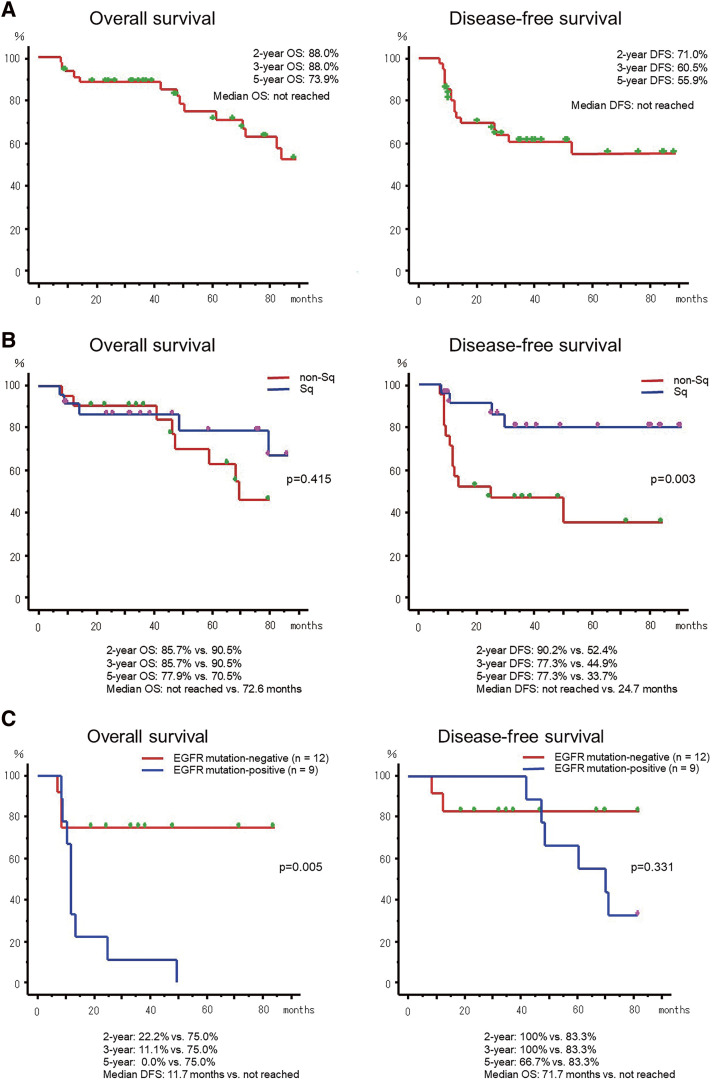
Kaplan–Meier curves of OS and DFS. (**A**) OS and DFS in the entire cohort. (**B**) OS and DFS in the Sq and non-Sq cohorts. (**C**) OS and DFS according to EGFR mutation status in the non-Sq cohort (exploratory analysis). OS, overall survival; DFS, disease-free survival; EGFR, epidermal growth factor receptor; Sq, squamous

After a median follow-up period of 49.2 months (range: 3.2–120.5 months), the median OS for the entire cohort was not reached. The 2-, 3-, and 5-year OS rates were 88.0%, 88.0%, and 73.9%, respectively. The median DFS for the entire cohort was not reached, and the 2-, 3-, and 5-year DFS rates were 71.0%, 60.5%, and 55.9%, respectively (**Fig. 2A**). The 5-year OS rate was 77.9% in the Sq cohort and 70.5% in the non-Sq cohort, with no statistically significant difference between the 2 cohorts (p = 0.415). The median OS was not reached in the Sq cohort and was 72.6 months in the non-Sq cohort. In contrast, the 5-year DFS rate was significantly higher in the Sq cohort than in the non-Sq cohort (77.3% vs. 33.7%, p = 0.003). The median DFS was not reached in the Sq cohort, whereas it was 24.7 months in the non-Sq cohort. Among the 45 patients who underwent surgery, 24 patients achieved nodal downstaging (ypN0), whereas 21 patients had persistent nodal disease (ypN1–2). Patients with ypN0 status tended to have better survival outcomes than those with residual nodal disease. The 5-year OS rate was 90.2% in ypN0 patients and 59.3% in patients with persistent nodal disease (p = 0.046).

### Exploratory analysis of EGFR mutation status in the non-Sq cohort

As an exploratory study, EGFR mutation analysis was retrospectively performed on 21 of 23 patients in the non-Sq cohort who underwent surgical resection (2 of whom had insufficient tumor tissue for molecular analysis). Of these 21 patients, 9 (42.9%) had EGFR mutations (exon 19 deletion in 7 patients and L858R point mutation in 2 patients), while 12 (57.1%) were EGFR mutation-negative. The 3-year OS rate was significantly lower in patients with EGFR mutations than in those without EGFR mutations (11.1% vs. 75.5%, p = 0.005; **Fig. 2C**). The median DFS was 11.7 months in the EGFR mutation–positive group, whereas it was not reached in the EGFR mutation–negative group. However, the 3- and 5-year OS rates were similar between the EGFR mutation–positive and EGFR mutation–negative groups. These results likely reflect the significant benefit of administering EGFR-tyrosine kinase inhibitor (TKI) therapy after relapse in the EGFR mutation–positive subgroup. Of the 9 EGFR mutation–positive patients, 8 (88.9%) experienced a relapse during follow-up. All 8 patients who relapsed received EGFR-TKI therapy as first-line treatment for relapse, with an objective response rate of 87.5% (7 of 8). The median time from relapse to progression while on EGFR-TKI therapy was 18.3 months. Some patients who progressed while on first-generation TKI therapy subsequently received osimertinib. Additionally, although EGFR mutations are more frequently observed in adenocarcinoma, the inclusion of a heterogeneous non-Sq cohort may also have influenced the observed mutation frequency.

### Patterns of recurrence

During the follow-up period, recurrence occurred in 17 of 45 patients (37.8%) who underwent surgery. The recurrence rate was significantly higher in the non-Sq cohort than in the Sq cohort (61.9% vs. 16.7%, p = 0.002) (**Table 4**). Among patients who experienced recurrence, the distribution of recurrence patterns did not differ significantly between the 2 cohorts. Distant metastases were the most common pattern of recurrence in both cohorts (Sq: 12.5%, non-Sq: 47.6%). Locoregional recurrence occurred in 3 patients (12.5%) in the Sq cohort and 6 patients (28.6%) in the non-Sq cohort. Among the 9 patients with EGFR-mutated tumors, 8 patients received EGFR-TKI therapy after recurrence, whereas 1 patient received platinum-based chemotherapy.

**Table 4:** Recurrence patterns and oncologic outcomes after surgery

	Sq (n = 24)	Non-Sq (n = 21)	p Value
No recurrence	20 (83.3%)	8 (38.1%)	*0.002*
Recurrence overall	4 (16.7%)	13 (61.9%)	
Local recurrence	3 (12.5%)	6 (28.6%)	
Distant recurrence	3 (12.5%)	10 (47.6%)	
Site of distant recurrence			
Brain	0 (0%)	5 (23.8%)	
Liver	1 (4.2%)	0 (0%)	
Bone	2 (8.3%)	3 (14.3%)	
Lung	2 (8.3%)	4 (19.0%)	

p values under 0.05 are provided in italic.

Sq, squamous; non-Sq, non-squamous cell carcinoma

## Discussion

The role of surgery after induction chemoradiotherapy for stage IIIA-N2 NSCLC has been controversial. van Meerbeeck et al. conducted a phase III trial involving 579 patients with pathologically confirmed stage IIIA-N2 NSCLC to compare surgery and radiotherapy after induction chemotherapy.^17)^ The median OS in the surgery group was 16.4 months, with a 5-year survival rate of 15.7% and a perioperative mortality of 4%. The authors concluded that surgical resection did not improve outcomes compared with definitive CRT. Similarly, Albain et al. reported results from the INT 0139 trial, which randomized patients to either surgery or additional radiotherapy following induction CRT.^4)^ The median OS was 23.6 months in the surgery group and 22.2 months in the radiotherapy group, with no significant difference. However, treatment-related mortality was significantly higher in patients who underwent pneumonectomy (25.9%) compared to lobectomy (1.0%). Exploratory analysis revealed that lobectomy improved OS compared to matched nonsurgical patients (33.6 vs. 21.7 months, p = 0.002), whereas pneumonectomy did not show a survival benefit. In our study, neoadjuvant CRT was completed in all 48 patients, and 45 patients (93.8%) underwent surgery with no treatment-related deaths during either the induction or surgical phases. The median OS for patients who underwent surgery was 90.4 months, with 3- and 5-year survival rates of 88.0% and 73.9%, respectively. These results compare favorably with previous trimodality studies. Importantly, pneumonectomy was avoided in all cases through the use of bronchoplasty or pulmonary artery reconstruction in 10 patients (22.2%), which likely contributed to the absence of surgery-related mortality and the favorable outcomes.

Previous studies have demonstrated that chemotherapeutic efficacy differs according to histological subtype in NSCLC. Cisplatin plus vinorelbine has shown efficacy in Sq,^13,18)^ whereas cisplatin plus pemetrexed has demonstrated superior outcomes in non-squamous histology.^19)^ However, no previous prospective study has systematically compared neoadjuvant CRT outcomes based on histology-tailored chemotherapy regimens. In our study, we prospectively assigned patients to receive either cisplatin–vinorelbine (Sq cohort) or cisplatin–pemetrexed (non-Sq cohort) with concurrent 50 Gy radiotherapy. The Sq cohort demonstrated markedly superior radiological response rates (92.0% vs. 30.4%, p <0.001), pCR rates (45.8% vs. 4.8%, p = 0.002), and long-term DFS (5-year DFS: 77.3% vs. 33.7%, p = 0.003). The median OS in both cohorts (Sq: not reached; non-Sq: 72.6 months) was clearly superior to that reported in previous neoadjuvant CRT studies (13–32 months).^14,19,20)^ The superior outcomes in the Sq cohort likely reflect both the intrinsic radiosensitivity of Sq and the synergistic effect of cisplatin–vinorelbine with concurrent radiotherapy. In contrast, the non-Sq cohort showed more modest pathological response and DFS, which may be attributable to several factors including molecular heterogeneity, differential radiosensitivity, and the presence of EGFR mutations in a substantial proportion of cases.

Our exploratory analysis revealed that among 21 patients in the non-Sq cohort who underwent surgery, 9 patients (42.9%) harbored EGFR mutations. The 3-year DFS rate was significantly inferior in EGFR mutation–positive patients compared with EGFR mutation–negative patients, with a median DFS of 11.7 months versus not reached, respectively. However, OS was comparable between the 2 groups, likely reflecting the substantial efficacy of salvage therapies administered after recurrence, particularly EGFR-TKIs. These findings should be interpreted with caution. The poorer DFS observed in EGFR-mutated tumors does not necessarily indicate that induction chemoradiotherapy is ineffective in this subgroup. Rather, it may reflect the distinct biological behavior of EGFR-mutated NSCLC, which is characterized by a higher propensity for distant recurrence. Recent studies have suggested that perioperative EGFR-TKI strategies may provide improved outcomes in EGFR-mutant resectable NSCLC, with neoadjuvant osimertinib demonstrating superior pathological response rates and event-free survival compared with chemotherapy.^21)^ Therefore, perioperative EGFR-TKI therapy may represent an alternative strategy for EGFR mutation–positive patients. It should be emphasized that the EGFR mutation analysis in this study was not prespecified and represents a post-hoc exploratory analysis based on a small number of patients. Accordingly, these findings should be considered hypothesis-generating and require validation in larger prospective studies.

In this study, preoperative CRT was well tolerated in both cohorts, and treatment compliance was high. Regarding grade 3–4 adverse events, the incidence of neutropenia, nausea, and esophagitis was higher in the Sq cohort. However, all patients completed the planned preoperative CRT; no patient was unable to undergo surgery due to side effects, and no deaths were due to postoperative complications. Therefore, this treatment is considered to be a tolerable method. The tolerability of induction chemoradiotherapy in this study was acceptable, despite a relatively high incidence of grade 3/4 adverse events. All 48 patients completed the planned induction therapy, and toxicity did not prevent surgical resection in patients deemed resectable after restaging. Importantly, there were no treatment-related deaths in either the induction or surgical treatment groups. Although 25 patients (52.1%) required a temporary interruption of radiation therapy due to hematologic toxicity, all patients ultimately completed 50 Gy. This high treatment completion rate compares favorably with many previous studies of trimodality radiation therapy and suggests that concurrent chemoradiotherapy and surgery, even with relatively intensive regimens, is feasible with appropriate supportive care and toxicity management.

Radiation dose selection and rationale: For induction chemoradiotherapy, a total dose of 50 Gy was administered in 25 fractions. This dose is lower than the 60–66 Gy commonly used in definitive chemoradiotherapy. This dose selection was based on our institutional experience and published data suggesting that 50 Gy can effectively improve tumor downstaging while keeping surgical complications within acceptable limits.^13,14)^ The rationale for using low-dose radiation in neoadjuvant therapy is to achieve sufficient tumor shrinkage while minimizing fibrosis and tissue inflammation, which may complicate subsequent surgery. The choice between 50 Gy and higher doses (e.g., 54–60 Gy) in neoadjuvant therapy is currently under investigation. Higher doses may improve tumor control but increase the risk of postoperative complications, particularly pulmonary and cardiac toxicity. Our findings suggest that 50 Gy with concurrent chemotherapy provides a reasonable balance between oncological efficacy and surgical safety, but prospective randomized trials are needed to clearly establish the optimal radiation dose for neoadjuvant chemoradiation.

This study has several important limitations that should be acknowledged. First, it was a single-center observational study conducted at a tertiary care medical institution with extensive experience in thoracic surgery and multidisciplinary care. The generalizability of our findings to other institutions with different patient populations, surgical expertise, and supportive care resources may be limited. Multicenter validation would strengthen the external validity of our results. Second, at the time of its initiation in 2010, this study was not registered in a public clinical trial registry. While prospective trial registration is now standard practice and required for publication in most academic journals, it is important to note that mandatory registration requirements were not uniformly implemented in Japan at the time, particularly for observational studies. This study was conducted under IRB supervision, and written informed consent was obtained from all participants. The primary and secondary endpoints were defined in an IRB-approved protocol. Third, the sample size was relatively small, which may have reduced the statistical power to detect differences between subgroups. The small number of patients in the exploratory subgroup analyses, especially the EGFR mutation analysis, significantly limits the robustness and generalizability of these findings. Fourth, programmed death-ligand 1 expression status and contraindications to ICI therapy were not assessed in this study cohort. Patient enrollment in this study began in 2010, when ICIs were not yet available for the treatment of NSCLC. The lack of programmed death-ligand 1 data makes it difficult to contextualize the study findings within the contemporary treatment landscape and precludes assessment of whether this patient population would have benefited from ICI-based therapy if it had been available at the time. Therefore, the present cohort should primarily be interpreted as historical benchmark data for outcomes following induction chemoradiotherapy followed by surgery. Nevertheless, in contemporary clinical practice, a subset of patients remains ineligible for ICI-based therapy due to contraindications such as autoimmune disease, severe immunosuppression, or organ transplantation. Although perioperative immunotherapy has recently changed the treatment landscape of resectable NSCLC, the strategy described in this study may still remain relevant for selected patients who are not eligible for ICI-based therapy. Fifth, this study spanned a long period (2010–2025), during which diagnostic technologies, imaging modalities, and supportive care strategies evolved. However, all patients had pathologically confirmed N2 disease, and the diagnostic approaches (EBUS-TBNA, mediastinoscopy, and VATS) did not differ significantly between cohorts. Sixth, EGFR mutation analysis was performed retrospectively as a post-hoc exploratory study and was not prespecified in the original study protocol. Given the small sample size, retrospective nature of the study, and lack of prior specification, these findings should be viewed as hypothesis-generating rather than definitive. Prospective validation in larger cohorts is needed to confirm the prognostic value and predictive ability of EGFR mutation status in preoperative chemoradiation.

## Conclusion

This study demonstrated that neoadjuvant CRT followed by surgery is highly feasible in carefully selected patients with stage IIIA-N2 NSCLC. This provides valuable benchmark data for a patient population in which ICIs are unavailable due to contraindications. For Sq and EGFR mutation–negative non-Sq, histology-specific neoadjuvant CRT followed by surgery resulted in favorable outcomes. For EGFR mutation–positive non-Sq, neoadjuvant CRT followed by surgery resulted in poor DFS, and the use of TKIs before surgery may be a better approach. Future large-scale, multicenter studies may be warranted to validate our findings.

## Declarations

### Data availability

The datasets used and/or analyzed during the current study are available from the corresponding author on reasonable request.
